# Effect of Simulated Climate Warming on the Ectomycorrhizal Fungal Community of Boreal and Temperate Host Species Growing Near Their Shared Ecotonal Range Limits

**DOI:** 10.1007/s00248-017-1044-5

**Published:** 2017-07-25

**Authors:** Joanna Mucha, Kabir G. Peay, Dylan P. Smith, Peter B. Reich, Artur Stefański, Sarah E. Hobbie

**Affiliations:** 1 Institute of Dendrology, Polish Academy of Sciences, Kórnik, Poland; 2 Department of Biology, Stanford University, Stanford, CA 94305 USA; 3 University of California, California Institute for Quantitative Biosciences, Berkeley, CA USA; 4 Department of Forest Resources, University of Minnesota, St. Paul, MN USA; 5 Western Sydney University, Hawkesbury Institute for the Environment, Penrith, NSW Australia; 6 Department of Ecology, Evolution and Behavior, University of Minnesota, St. Paul, MN USA

**Keywords:** Belowground communities, Ecotonal boundary, Temperature increase, Ectomycorrhizal fungi

## Abstract

Ectomycorrhizal (ECM) fungi can influence the establishment and performance of host species by increasing nutrient and water absorption. Therefore, understanding the response of ECM fungi to expected changes in the global climate is crucial for predicting potential changes in the composition and productivity of forests. While anthropogenic activity has, and will continue to, cause global temperature increases, few studies have investigated how increases in temperature will affect the community composition of ectomycorrhizal fungi. The effects of global warming are expected to be particularly strong at biome boundaries and in the northern latitudes. In the present study, we analyzed the effects of experimental manipulations of temperature and canopy structure (open vs. closed) on ectomycorrhizal fungi identified from roots of host seedlings through 454 pyrosequencing. The ecotonal boundary site selected for the study was between the southern boreal and temperate forests in northern Minnesota, USA, which is the southern limit range for *Picea glauca* and *Betula papyrifera* and the northern one for *Pinus strobus* and *Quercus rubra*. Manipulations that increased air and soil temperature by 1.7 and 3.4 °C above ambient temperatures, respectively, did not change ECM richness but did alter the composition of the ECM community in a manner dependent on host and canopy structure. The prediction that colonization of boreal tree species with ECM symbionts characteristic of temperate species would occur was not substantiated. Overall, only a small proportion of the ECM community appears to be strongly sensitive to warming.

**Electronic supplementary material:**

The online version of this article (doi:10.1007/s00248-017-1044-5) contains supplementary material, which is available to authorized users.

## Introduction

Boreal forests are expected to experience some of the most extreme increases in air temperature as a result of climate change. An increase of 1.5 °C has already been observed in the boreal zone [1], with additional warming of 4–6 °C expected in the next century [2]. As climatic conditions (predominantly temperature and precipitation) can influence the geographical distribution of plant species [3], future warming is likely to significantly impact the biology of resident species. Higher temperatures may prolong the growing season, extend plant water and nutrient uptake, and lead to changes in above and below ground net primary production and litter quality [4]. Increased rates of decomposition in boreal forests may enhance the loss of carbon from previously frozen soils and affect plant growth through increased mineralization of organic nutrients [5]. These environmental changes may be of special importance to boreal plant species that have evolved adaptations to reduced nutrient cycling rates that include the allocation of relatively more biomass to fine roots [6] and the production of absorptive fine roots with a relatively thick root cortex suitable for colonization by ectomycorrhizal fungi [7]. Roots associated with symbiotic microbes are able to optimize nutrient absorption under diverse environmental conditions [8]. Ectomycorrhizal fungi (ECM) are responsible for the majority of nutrient uptake in boreal tree species [9] and constitute a direct link between the above- and belowground components of boreal ecosystems. Thus, the loss or gain of mycorrhizal symbionts may affect host tolerance to changing climatic conditions [10], especially at the trailing, southern edges of boreal plant ranges. Although the geographical range of boreal species is expected to recede northwards while the range of temperate species is expected to expand, boreal species still occupy and function, despite decreased growth, in the transition zone between temperate and boreal species [11]. Given the interaction that occurs in the transition zone, limited knowledge exists pertaining to the response of the ectomycorrhizal community to climate change and how it might mediate changes in the community structure of boreal forests.

The response of ectomycorrhizal fungi to climate change will be affected by several factors that may alter the strength of the above/belowground linkage. Modified climatic conditions can affect mycorrhizal fungi directly and also through effects on the symbiotic partner, which may indirectly have an effect on the mycorrhizal fungal community. Mycorrhizal colonization is dependent on carbon allocation to roots by host plants, with up to 20–30% of seedling assimilate being allocated to ECM fungi [12]. This carbon source is directly used for the growth of extensive mycelial networks [13]. Tree species growing in the nitrogen-limited environment of boreal forests have higher relative allocation of tree photosynthate to their microbial symbionts compared to trees growing in fertile habitats [14], which in turn can improve tree N nutrition by means of fungal-mediated nutrient uptake from soil [15]. Patterns of carbon allocation within tree change significantly with forest structure, peaking in belowground with canopy closure, where leaf area index and nutrient demand are maximal [16, 17]. As boreal tree species show reduced photosynthesis with warming at the boreal-temperate ecotone, and temperate trees increased rates [18], this may signal reduced flow of carbohydrate to ECM fungi of boreal species under warmer climate conditions, resulting in the promotion of less carbon demanding species [19]. Previous research has demonstrated that carbon productivity increases ectomycorrhizal diversity [20, 21]. Therefore, temperate species growing near their northern range limit may support a higher diversity of ECM or investment in extramatrical hyphae due to the increased belowground carbon allocation that may occur due to increased regional temperature.

Fungi can grow under a wide range of temperatures [22]. Reduced species dissimilarity, however, may have an adverse effect on an ecosystem due to antagonistic interactions [23], as was reported for ECM isolates colonizing host plant roots under increased competition among fungi [24]. Thus, increasing the taxonomic, morphological, and functional diversity of ECM fungi may allow host plants to tolerate changes in environmental conditions in the broader geographical range, especially under warming and/or drying conditions [25]. Results of previous studies on the effects of warming on ECM symbiosis have been somewhat mixed. Warming experiments have been reported to cause changes in biomass and community composition of ECM fungi observed in several studies in the arctic tundra [26, 27] and boreal forests [28], as well as in growth chamber studies [29, 30]. Air temperature, however, has been reported to have a minimal influence on the community composition of ECM fungi at a regional scale [31] or on the richness of ECM fungi over substantial gradients including temperature [32, 33]. Most studies, however, have focused on a single species, which may obscure important effects of warming as species replacement occurs along range boundaries of temperate and boreal taxa. Including a greater number of plant species in studies may be important since host identity can have a strong influence on species richness and the community composition of ECM fungi [34–36]. Meta-analyses of many plant species have indicated that a lower phylogenetic diversity of ECM taxa exists in subarctic and tropical zones [37, 38], implying that an environmental filtering occurs at both high and low temperatures. Climate and host identity parameters, however, are confounded in studies over large geographic areas, leaving the primary drivers of these patterns unclear.

In order to determine how ectomycorrhizal communities respond to climate change, we assessed the composition of ectomycorrhizal fungi on seedlings in a long-term temperature manipulation experiment (B4WarmED) [18] in northern Minnesota at the ecotone between temperate and boreal forests. Root communities of planted juveniles of deciduous and conifer species representative of the boreal and temperate zones were surveyed using next-generation DNA sequencing. The sampling also included a canopy manipulation in order to determine how factors such as light availability might modify ECM fungal community response to global climate change (GCC). Previous research in this experiment demonstrated that in response to elevated temperatures (simulated climate warming), temperate species at their northern range limit increased their photosynthesis and growth while boreal species at their southern range limit decreased their photosynthesis and growth [18]. We hypothesized that, correspondingly, ECM fungal richness would increase in temperate hosts in the warmed plots and decrease in boreal hosts. In addition, we hypothesized that ECM fungal community composition in the warmed plots would be dominated by fungi found on temperate host plants. Lastly, we expected that richness would increase in open canopy plots due to increased belowground carbon allocation.

## Material and Methods

### Research Site and Sampling

The study site is located in an upland forest at the Cloquet Forestry Center in Cloquet, Minnesota USA (46°31′N, 92°30′E, 386 a.s.l.). The site has a mean annual temperature of 4.5 °C and a mean annual precipitation of 807 mm. The B4Warmed project (Boreal Forest Warming at an Ecotone in Danger) is located in a natural mixed aspen-birch-fir stand (approx. 40–60 years old) [18]. Soils are classified as the Omega-Cloquet-Cromwell association and consist of a sandy loam with a gravelly underlying material. Experimental plots in the B4Warmed site were planted with nursery-grown seedlings established from local seed of five boreal (*Picea glauca*, *Pinus banksiana*, *Abies balsamifera*, *Betula papyrifera*, and *Populus tremuloides*) and five temperate (*Acer sacharum*, *A. rubrum*, *P. strobus, Quercus macrocarpa*, and *Q. rubra*) tree species in the native ground vegetation. Temperature treatments included a treatment control (inactive heating cables and lamps) and warming treatments of +1.7 and +3.4 °C above ambient temperature [39]. The differences in temperature were +1.7 and +3.4 °C belowground at the depth of 10 cm and +1.82 and +3.45 °C aboveground for the plant canopies [39]. Plants were heated both aboveground with overhead infrared heat lamps and belowground with buried heating cables at the depth of 10 cm. Seedlings were transplanted into the plots in the spring of 2008. The warming treatments were applied continuously for 24 h/day for approximately 8 months/year from about March to November starting in the spring of 2009. Temperature treatments were administered in a fully factorial design with two treatments that were designed to replicate understory (~5–10% of full light) and open (~40–60% of full light) conditions, similar to what might occur after timber harvest. Greater technical details regarding the B4Warmed Project are available in Rich et al. [39].

In order to characterize the ECM fungal community, roots were sampled from four species growing in the B4WarmED experimental plots. The sampling included two boreal (*P. glauca* and *B. papyrifera*) and two temperate (*P. strobus* and *Q. rubra*) tree species. The chosen species represent conifer and angiosperm species from each zone since they differ in anatomy and physiology and may respond differently to warming [see 40]. Roots were collected in March, 2012 during destructive harvests of trees, directly from intact root systems of 2 to 7 individuals of each species. As a result, the host identity was known. The sampling included 3 temperature treatments × 2 canopy treatments × 4 species × 2 to 7 seedlings which provided a total *N* = 129. For each individual, 15 ECM root tips were removed from each individual under a dissecting scope from a random collection of different root fragments gently washed in water. Bulked ECM root tips were placed in tubes filled with 300 μL of 2× CTAB buffer [41] and frozen at −80 °C until further processing. Four to seven seedlings for each unique treatment (species × canopy × temperature) were taken from different plots.

### DNA Extraction, PCR Amplification, and Sequencing

Root tips were homogenized using three 3-mm zirconia beads in a tissue homogenizer (FastPrepTM FP120, Thermo Savant, Qbiogene, Carlsbad, CA) at a speed setting of 5.5 for 45 s. DNA was then extracted from the homogenized samples using a Qiagen Tissue Kit (Qiagen, Valencia, CA, USA) according to the manufacturer’s protocol (Qiagen, Valencia, CA, USA), with the addition of a preliminary chloroform extraction as described by Peay et al. [41]. PCR reactions were performed in a total volume of 25 μl consisting of 0.125 μl of HotStarTaq polymerase (5 μ/μl, Qiagen, Valencia, CA, USA), 5 μl of 5× PCR buffer, 0.5 μl of each of the 10 μM the primers, 5 μl of 10 mM dNTP, and 1 μl of template DNA. The internal transcribed spacer (ITS) region of the nuclear ribosomal RNA genes was amplified using the fungal specific primer pair ITS1f [42] and ITS4 [43]. The primers were modified for next-generation sequencing on the 454 platform by adding an A adapter to the 5′-CCATCTCATCCCTGCGTGTCTCCGACTCAG-3′ and a 10 base pair barcode to the 5′ end of the ITS1F primer and the 454 B adapter 5′-CCTATCCCCTGTGTGCCTTGGCAGTCTCAG-3′ to the 5′ end of the ITS4 primer. Cycling conditions were as follows: after an initial denaturation step at 95 °C for 1 min, PCR was cycled 30 times at 94° for 30 s, at 52 °C for 30 s, at 68 °C for 1 min, followed by a final extension at 68 °C for 7 min. Negative controls were included alongside all DNA extractions, and PCR reactions provided clean products as determined by gel electrophoresis. Successful PCR products were purified using Agencourt AMPure XP (Beckman Coulter, Brea, California) following the manufacturer’s instructions and quantified using the Quant-iT dsDNA HS Assay Kit (Life Technologies, Carlsbad, California). Samples were pooled in equimolar concentrations and sequenced at the Duke University ISGP Sequencing Facility on a 1/8 partition of a 454 Life Sciences FLX Titanium Shotgun plate (454 Life Sciences).

### Bioinformatics

Qiime v. 1.7.0. [44] was used to process the resulting 454 dataset. For quality control, all reads with more than 1.5 barcode error, no valid primer or DNA tag, or any sequences with ambiguous bases or those that were shorter than the length threshold of <350 bp, were removed. After quality control, the dataset was reduced from 72,168 to 41,637 reads. The remaining sequences were assigned to operational taxonomic units (OTUs) with the USEARCH algorithm [45] using a 97% sequence identity and default parameters for minimum overlap, matches, mismatches, and gap penalties. A taxonomic designation was assigned to a representative sequence from each OTU using the BLAST option in QIIME’s *assign_taxonomy.py* script against the UNITE v6 database [46; http://unite.ut.ee/repository.php] with dynamic clustering threshold accessed on 30 December 2014. The OTU table was filtered to include only confirmed or potential ECM taxa based on Tedersoo et al. [47] and Branco et al. [48]. After filtering for ECM fungal taxa, we rarefied the OTU table to 50 sequences per sample using the *multiple_rarefaction.py* script, a level that maximized sample retention while still providing adequate sequencing depth. Diversity indices (chao1, observed species, Shannon diversity, Simpson diversity, Shannon and Simpson evenness, and phylogenetic diversity) were calculated at a depth of 50 sequences using the *alpha_diversity.py* script in Qiime.

### Statistical Analysis

A general linear model (GLM) was used to test whether or not *temperature*, *canopy* manipulations, and *host species* affected fungal diversity metrics. The statistical model included all main effects and pairwise interactions of main factors. A three-way interaction, however, was not included with all of the studied factors in our model due to the lack of appropriate replication. A t-statistic was applied to estimate significance of fixed effects (*P* = 0.05), and pairwise differences between treatment groups were assessed with Tukey’s honest significant difference (HSD) test.

Despite the variability in PCR efficiency [49, 50] and ITS copy number [51, 52], which may vary across taxa, quantitative sequence data are more accurate than presence-absence data in estimating community differences [53, 54]. Similarly, Amend et al. [55] found that relative abundance across samples was also a reliable measure. For this reason, we based our analyses of community composition on sequence abundance data. To test which factors were significant drivers of variability in ECM fungal community composition, *temperature*, *canopy*, and *host*, and their interaction, were used as predictor variables for multispecies generalized linear models (GLM) applied by means of the R package *mvabund* [56]. In contrast to dissimilarity-matrix-based methods, *mvabund* avoids confounding location with dispersion effects, which can inflate type 1 and type 2 errors [57]. The “*negative.binomial*” was used as suggested for multivariate abundance models based on count data [58]. Multiple tests were corrected for using the *p.adjust*(*method =* “*fdr*”) function in R’s base package. *P* values were determined based on 1000 permutations. Redundancy analysis (RDA) in the VEGAN package of R (R Core Development TEAM, 2007) was used to visualize the potential impact of temperature treatment on the fungal community.

## Results

### Taxonomic Coverage

A total of 41,517 sequences were generated and assigned to 358 OTUs (Table S1). Within the OTUs, 292 (48.5% of sequences) matched other fungi, 84 (51.4% of sequences) ECM, 2 (0.005% of sequences) were unidentified fungi, and 7 (0.075% of sequences) received no blast hit. Among the OTUs, 174 (77% of sequences) were classified as *Basidiomycota* and 130 (22% of sequences) belonged to the *Ascomycota* (Tables S1, S2, S3). The *Zygomycota* and *Glomeromycota* were represented by 23 (0.45% of sequences) and 22 (0.95% of sequences) OTUs, respectively. The most frequently encountered ECM taxa belonged to the genera *Lactarius*, *Laccaria*, *Tomentella*, *Russula*, *Hebeloma*, and *Tuber* (Table 1), and the greatest number of ECM OTUs were found in *Inocybe* and *Tomentella* (OTUs 16 and 15, respectively). The most common ECM fungal taxa were found in less than 25% of the samples.

**Table 1:** Percentage of the occurrence of the most frequent ECM fungal taxa in different study condition (canopy, temperature and species)

OTUs	BLAST % match	GenBank match	Total	Open canopy												Closed canopy											
				*P. glauca*			*B. papyrifera*			*P. strobus*			*Q. rubra*			*P. glauca*			*B. papyrifera*			*P. strobus*			*Q. rubra*		
				A	1.7 °C	3.4 °C	A	1.7 °C	3.4 °C	A	1.7 °C	3.4 °C	A	1.7 °C	3.4 °C	A	1.7 °C	3.4 °C	A	1.7 °C	3.4 °C	A	1.7 °C	3.4 °C	A	1.7 °C	3.4 °C
*Lactarius sp*	100	UDB005368	22	13	3	3	3	3	0	6	3	3	3	0	0	3	3	3	3	3	3	16	0	3	6	3	10
*Lactarius aurantiacus*	100	KF432974	18	12	4	4	4	4	0	4	0	4	0	0	4	4	0	4	4	4	4	16	0	4	12	0	8
*Laccaria laccata*	100	KC152138	17	4	0	13	0	0	4	4	0	8	4	0	0	0	13	8	8	4	0	4	4	13	0	8	0
*Tomentella botryoides*	100	KC952675	17	4	4	0	4	4	0	4	0	0	4	0	0	13	4	9	9	0	4	13	0	0	9	9	4
*Russula delica*	100	UDB016008	12	6	6	0	0	0	0	6	0	0	0	6	0	18	6	6	6	12	0	6	6	6	6	0	6
*Tomentella muricata*	100	UDB003303	12	0	6	0	0	0	0	0	0	0	0	0	0	0	0	12	6	0	6	0	6	0	0	0	6
*Hebeloma helodes*	100	HQ179675	12	0	0	13	0	0	6	0	13	0	13	13	0	0	0	0	0	13	0	6	0	0	0	19	6
*Tuber sp*	100	AY634174	12	6	13	13	13	0	0	13	6	6	6	0	0	0	0	0	0	6	0	0	6	0	6	6	0
*Tomentella coerulea*	100	UDB003327	11	7	7	0	0	0	0	0	0	20	7	0	0	7	7	7	13	7	0	0	7	7	0	7	0
*Clavulina sp*	100	KC840629	9	8	8	0	0	0	0	8	0	8	0	0	8	0	8	0	8	0	23	0	0	0	8	15	0
*Tomentella sp*	100	JQ393140	9	0	0	08	0	0	8	8	8	0	8	0	8	0	8	0	17	0	0	0	17	0	0	8	0
*Wilcoxina mikolae*	100	HQ406818	7	0	0	10	0	0	10	10	0	10	0	0	0	10	20	10	0	0	0	10	0	10	0	0	0
*Tuber anniae*	100	HM485338	7	0	11	11	11	11	0	0	11	11	0	0	0	0	11	0	0	0	0	11	11	0	0	0	0
*Suillus flavidus*	100	UDB011444	6	0	13	0	0	0	0	38	25	25	0	0	0	0	0	0	0	0	0	0	0	0	0	0	0
*Inocybe lanatodisca*	100	JQ408760	6	0	0	0	0	0	13	0	0	0	0	0	13	0	13	0	0	13	0	0	13	0	13	13	13
*Inocybe nitidiuscula*	100	HQ604318	6	0	0	0	0	0	0	0	0	13	0	0	0	13	0	13	0	25	13	0	13	0	0	13	0
*Inocybe lacera*	100	AY750157	6	0	0	0	0	0	25	0	25	0	13	0	0	0	13	0	13	0	0	0	0	0	13	0	0
*Wilcoxina rehmii*	100	JQ989328	5	14	0	29	0	0	0	0	0	14	0	0	0	0	0	0	0	0	0	0	43	0	0	0	0
*Hydnobolites sp*	100	KF385313	5	14	0	0	14	0	0	0	0	0	0	0	0	14	29	14	0	0	0	0	14	0	0	0	0
*Amphinema sp*	100	DQ233799	4	0	17	17	0	0	0	0	0	17	0	0	0	17	17	17	0	0	0	0	0	0	0	0	0
*Clavulina sp*	100	EU862221	4	0	0	0	33	0	0	17	0	0	0	0	17	17	0	0	0	0	0	0	0	0	0	17	0
*Genea hispidula*	100	JX679370	4	17	0	0	0	0	0	17	0	0	0	0	0	0	0	0	0	17	0	0	0	0	17	33	0
*Amphinema sp*	100	AY969463	4	17	0	0	0	0	0	0	0	0	0	0	0	0	0	0	0	0	0	33	17	17	0	0	0
*Tomentella bryophila*	100	UDB001655	4	0	0	17	17	0	0	0	0	0	0	17	0	0	0	33	17	0	0	0	17	0	0	0	0
*Thelephora albomarginata*	100	UDB017321	4	20	0	0	20	0	0	0	0	20	0	0	0	0	0	0	0	20	0	0	0	0	0	20	0
*Russula cremeirosea*	100	EU819424	4	25	0	0	0	25	0	0	25	0	25	0	0	0	0	0	0	0	0	0	0	0	0	0	0
*Russula griseascens*	100	UDB016038	4	0	20	0	0	0	0	0	0	0	0	0	0	0	0	0	0	0	0	60	0	20	0	0	0

*A* ambient temperature, *1.7 °C* temperature above ambient, *3.4 °C* temperature above ambient

### Treatment Effects on Fungal Community Composition

Taxa that responded to experimental treatment were generalists (in reference to host compatibility) such as *Laccaria*, *Russula*, and *Tomentella* [59, 60], and we did not find increase of ECM fungal species with long exploration type characterized for warmer conditions. Two ECM taxa, *Lactarius* (OTU_2) and *Russula* (OTU_44), decreased in occurrence in the higher (3.4 °C) temperature treatment, in contrast to *Wilcoxina* (OTU_10 and OTU_24) and *Laccaria* (OTU_37), which increased in both higher temperature treatments (Table 1). *Lactarius* (OTU_2 and OTU_134) and several *Tomentella* (OTU_4, OTU_6 and OTU_59) and *Russula* (OTU_44) species were frequently present in closed canopy samples. *Tomentella*, *Clavulina*, and *Tuber* species exhibited diverse responses to the elevated temperature treatments.

Species that were potentially mycorrhizal or associated with mycorrhiza were identified among the other fungal taxa. The most frequently encountered species was *Phialocephala fortinii* (Table 2), which occurred in more than half of the samples. This taxon, however, was represented by only 3 OTUs in contrast to the more diverse *Mortierella* which was represented by 14 OTUs. The vast majority of taxa were found in less than 25% of the samples.

**Table 2:** Percentage of the occurrence of other fungal taxa in different study condition (canopy, temperature, and species)

	BLAST % match	GenBank match	total	Open canopy												Closed canopy											
				*P. glauca*			*B. papyrifera*			*P. strobus*			*Q. rubra*			*P. glauca*			*B. papyrifera*			*P. strobus*			*Q. rubra*		
				A	1.7 °C	3.4 °C	A	1.7 °C	3.4 °C	A	1.7 °C	3.4 °C	A	1.7 °C	3.4 °C	A	1.7 °C	3.4 °C	A	1.7 °C	3.4 °C	A	1.7 °C	3.4 °C	A	1.7 °C	3.4 °C
*Phialocephala fortinii*	100	AY033087	54	7	5	3	0	1	3	3	4	4	4	5	1	4	7	7	5	5	1	5	5	7	5	4	3
*Ascomycota* sp	100	DQ182439	30	5	2	10	5	2	0	7	5	5	5	5	5	0	5	2	2	10	2	2	5	7	0	2	5
*Helotiales* sp	100	HQ445006	25	9	0	3	6	3	3	9	0	6	11	3	3	3	3	0	6	3	9	3	3	3	9	3	3
*Cadophora finlandica*	100	AF486119	24	6	9	3	0	0	0	0	0	0	9	3	3	6	3	6	3	9	3	3	0	3	9	12	9
*Ilyonectria rufa*	100	AY677271	21	3	3	3	3	3	0	10	3	3	3	3	10	3	0	3	3	3	0	7	7	3	0	10	3
*Phialocephala fortinii*	99	AY033087	20	4	0	0	4	7	0	4	7	4	4	4	4	0	0	7	11	7	4	7	11	7	0	0	4
*Meliniomyces bicolor*	99	AJ430147	19	8	12	4	0	0	4	4	0	0	4	0	0	4	8	12	0	8	0	4	12	8	4	8	0
*Thelephoraceae* sp	100	EU625865	18	4	4	0	0	4	0	4	8	4	0	8	8	0	8	4	4	4	12	4	0	8	4	0	8
*Sebacinaceae* sp	100	HQ667874	17	4	4	4	4	4	8	8	4	8	4	8	8	0	8	4	4	0	0	0	0	0	4	4	4
*Pseudogymnoascus destructans*	100	KC171349	17	13	0	4	8	0	0	4	0	4	4	0	4	0	0	4	0	4	8	0	4	0	8	13	13
*Ascomycota* sp	100	GQ179993	17	9	9	4	4	9	9	9	0	0	4	0	0	0	0	0	0	0	9	4	0	0	9	9	9
*Gymnostellatospora japonica*	100	DQ117455	16	9	5	0	0	5	0	5	0	5	5	0	5	0	5	0	5	5	5	9	5	0	5	14	9
*Lachnella alboviolascens*	99	AY571048	16	0	0	0	5	0	3	0	5	0	5	0	5	5	9	9	0	9	0	0	14	5	0	5	14
*Ascomycota* sp	100	KC007187	15	0	0	0	0	0	9	5	10	10	0	0	5	0	5	0	5	0	0	5	14	14	0	14	14
*Leotiomycetes* sp	100	GU174363	12	0	6	6	0	6	0	0	6	0	19	6	6	6	0	0	0	6	0	13	13	0	6	0	0
*Thelephoraceae* sp	100	DQ233803	11	13	7	0	0	7	0	0	13	7	0	7	0	0	7	0	0	0	7	7	7	7	0	7	0
*Ascomycota* sp	99	HQ211601	11	0	0	20	0	7	7	0	7	13	7	0	0	0	0	7	0	7	7	0	7	0	7	7	7
*Clavulinaceae* sp	100	EF619643	10	0	0	0	0	0	0	0	7	0	0	7	14	0	7	0	7	7	0	0	14	0	14	7	14
*Ascomycota* sp	100	FJ904462	10	7	7	0	7	0	0	7	0	0	0	7	14	0	0	0	0	7	0	0	7	7	14	7	7
*Meliniomyces bicolor*	100	HQ445329	10	0	7	0	0	0	0	7	0	0	14	7	14	0	0	0	0	7	0	0	0	0	7	21	14
*Thelephoraceae* sp	100	DQ974796	10	0	7	7	7	0	0	7	0	0	7	0	7	0	0	21	7	0	7	0	0	0	0	14	7
*Ascomycota* sp	100	FM200472	9	0	0	0	0	0	0	8	0	0	8	0	8	8	0	0	15	0	0	15	8	0	0	15	15
*Mollisia* sp. *olrim132*	100	AY354269	9	0	0	0	0	0	0	0	0	0	23	15	8	0	0	0	8	0	0	0	0	0	8	31	8
*Mortierella* sp	100	JF461132	9	0	0	15	8	0	0	15	0	0	0	0	0	0	0	8	8	8	0	0	15	0	8	0	15
*Mortierella* sp	100	JQ666581	9	8	0	8	0	0	0	8	0	0	0	0	8	8	0	0	15	15	0	0	0	23	0	8	0
*Mycosphaerellaceae* sp	100	EU726287	9	8	0	8	0	0	8	8	8	25	0	0	0	0	0	8	0	0	0	8	0	17	0	0	0
*Sebacina* sp	100	UDB017059	8	0	0	0	9	0	0	9	0	9	18	0	0	0	9	0	9	0	0	9	0	9	9	9	0
*Helotiales* sp	100	AY627824	8	0	0	0	0	0	0	27	9	9	0	0	0	0	0	0	9	27	0	0	0	18	0	0	0
*Phialocephala fortinii*	99	AY033087	8	0	9	9	0	9	0	0	0	0	0	0	0	0	9	0	0	0	9	18	18	9	0	9	0

*A* ambient temperature, *1.7 °C* temperature above ambient, *3.4 °C* temperature above ambient

**Fig. 1:**
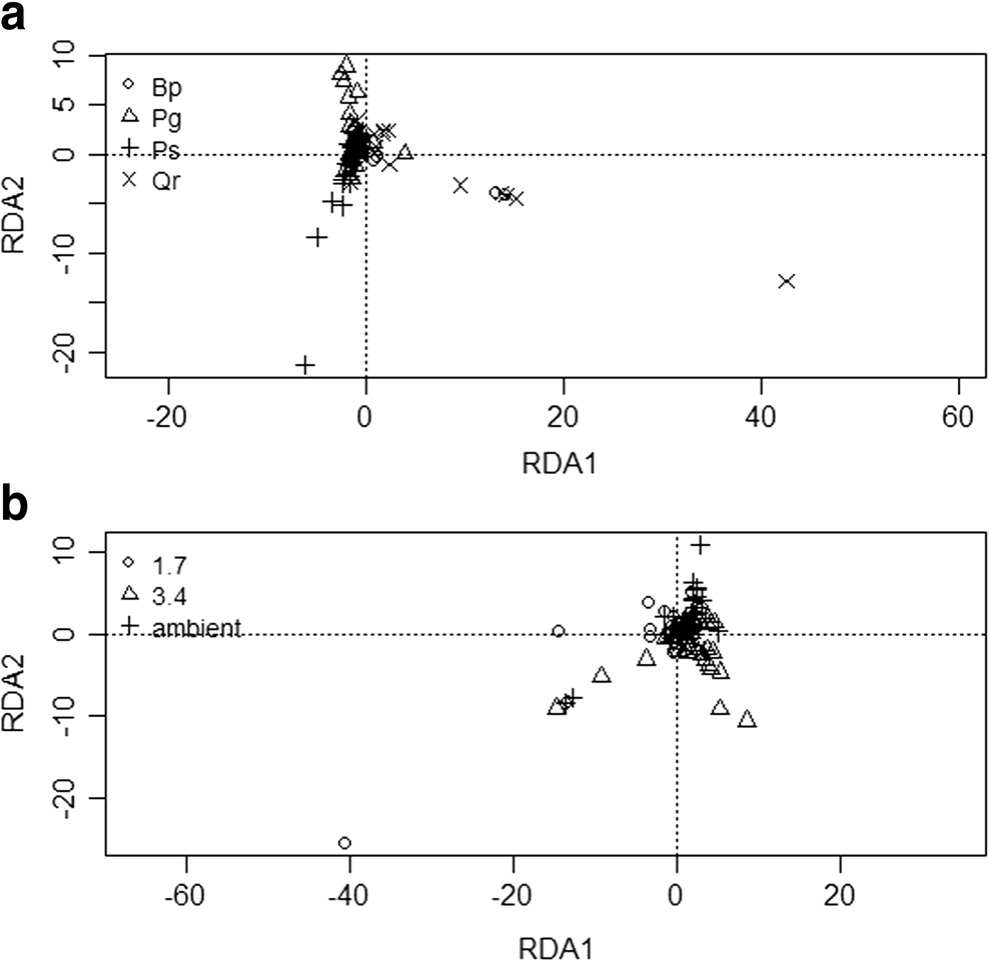
Ordination diagram illustrating the effects of species - *Picea glauca *(﻿Pg), *Betula papyrifera* (Bp), *Pinus strobus* (Ps), and *Quercus rubra *(Qr) (**a**)﻿ or temperature treatment (ambient, + 1.7 °C, and 3.4 °C) (**b**) based upon RDA analysis of ECM fungal communities

**Fig. 2:**
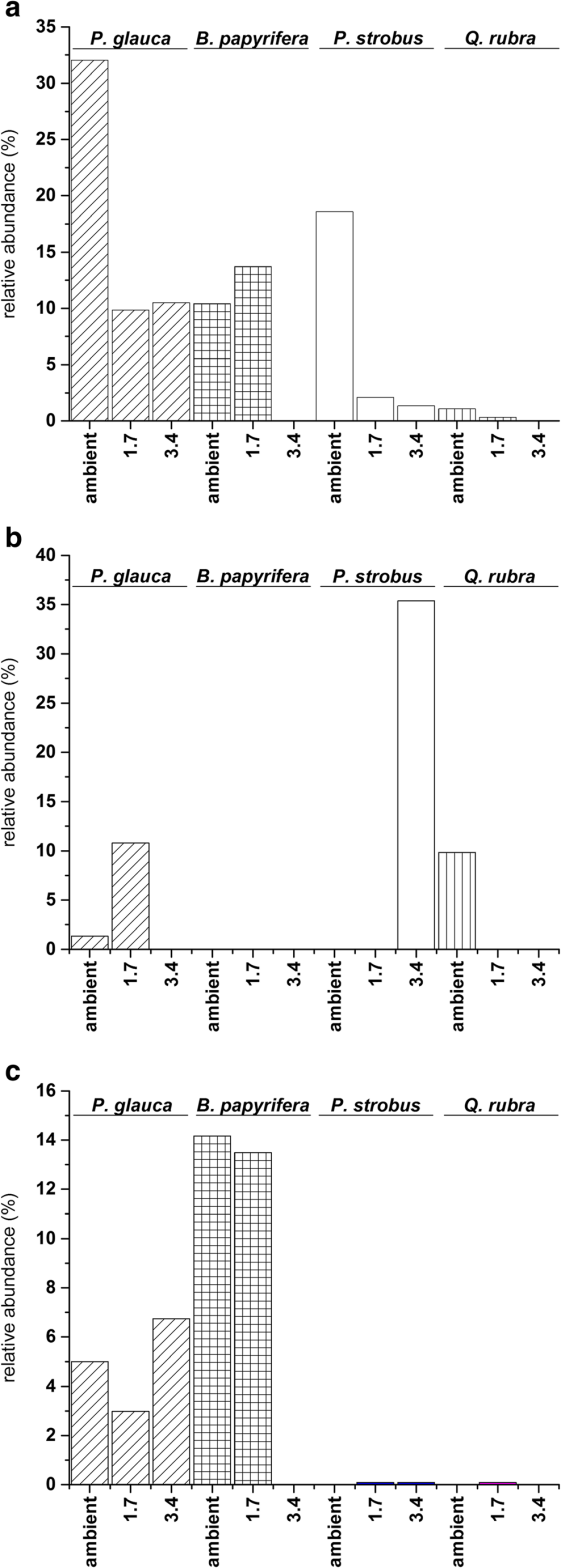
Relative abundance of selected OTUs under different experimental conditions. **a** *Russula delica* (OTU_44) abundance (species × temperature treatment). **b** *Tomentella coerulea* (OTU_6) in an open canopy. **c** *Tomentella coerulea* (OTU_6) in a closed canopy

Abundance data indicated that all of the experimental factors (host species, canopy, and temperature treatment) had a significant influence on ECM fungal community composition (Table 3 and Fig. 1) and other fungal taxa community composition (Table 4). Although we expected shifts in community composition across temperature, we did not confirm domination of ECM fungi characteristic to temperate species in the warlmer plots (those belong to long-distance exploration type). Four ECM fungal OTUs responded to treatments. Univariate tests confirmed that *Laccaria laccata* (OTU_37) and *Clavulina* sp. (OTU_30) varied among plant host species and temperature treatment, respectively. *L. laccata* was significantly more abundant (*P* = 0.020) on *P. abies*, *B. papyrifera*, and *P. strobus* than on *Q. rubra* roots, while *Clavulina* sp. was significantly more abundant (*P* = 0.014) in the ambient versus warm temperature treatments. A significant interaction (*P* = 0.019) between host species and temperature was demonstrated for the relative abundance of *Russula delica* (OTU_44) (Fig. 2a). *R. delica* was also more abundant at the ambient and lower, elevated temperature (1. 7 °C) on conifers and *B. papyrifera* roots but was barely detected on *Q. rubra* roots (relative abundance <1%). The occurrence of *L. laccata* revealed a contrasting response to temperature treatment and canopy. Specifically, it increased in abundance with elevated temperatures in the open canopy and the opposite trend occurred in the closed canopy (*P* = 0.002). The abundance of *Tomentella coerulea* (OTU_6) was affected by an interaction of canopy, temperature, and species (*P* = 0.020) (Fig. 2b, c).

**Table 3:** Multivariate test (nmanyglm) of ECM abundance taxon

Factor	Res. Df	Score	Pr(>score)
Canopy	120	131.6	0.001
Temperature treatment	121	253.1	0.001
species	123	358.3	0.001
Canopy × temperature treatment	109	117.5	0.001
Canopy × species	111	149.8	0.001
Temperature × species	114	292.8	0.001
Canopy × temperature treatment × species	103	41.4	0.001

**Table 4:** Multivariate test (anova.manyglm) of other fungal abundance taxon

Factor	Res. Df	Score	Pr(>score)
Canopy	131	390.4	0.01
Temperature treatment	132	705.9	0.01
species	134	1229.5	0.01
Canopy × temperature treatment	120	274.0	0.01
Canopy × species	122	307.6	0.01
Temperature × species	125	673.5	0.01
Canopy × temperature treatment × species	114	205.9	0.01

### Treatment Effects on Fungal Community Diversity

Although we expected that ECM richness would increase on temperate hosts and decrease on boreal hosts in response to temperature, ECM fungal richness (Chao1) was consistent for temperate and boreal species across temperature treatments (three-way ANOVA; host × temperature treatment interaction F = 1.521, d.f. = 6, *P* = 0.181) (Table 5). We did not observe dissimilarity across temperature treatments (three-way ANOVA; F = 0.452, d.f. = 2, *P* = 0.638) and host species (three-way ANOVA; F = 0.132, d.f. = 3, *P* = 0.941). Additionally, in contrast to our hypothesis that increased belowground carbon allocation in open plots would promote greater ECM richness, no difference in Chao richness was observed between canopy conditions (three-way ANOVA; F = 0.044, d.f. = 1, *P* = 0.834). The use of other diversity metrics (Shannon diversity and evenness, Simpson diversity and evenness, and phylogenetic diversity) gave similar results and did not show significant effects of host species, temperature treatment, or canopy individually or by their pairwise interaction on these indices and did not confirm our hypothesis. Analysis of other species revealed that the canopy treatment influenced richness and diversity, with the highest mean values obtained under closed canopy, opposite of what we expected (Table 6). Different patterns of richness for other fungi were observed in open vs. closed canopy treatments across temperature (canopy × temperature treatment interaction). Estimated richness was the highest under closed canopy treatments at 1.7 °C and both temperatures (1.7 and 3.4 °C) in an open canopy.

**Table 5:** General linear model of ECM fungal OTU richness based upon the rarefication of the observed species to 50 sequences

	DF	Richness metrics				Diversity metrics				Evenness metrics			
		Chao1		Observed species		Shannon		Simpson		Shannon		Simpson	
		F	P	F	P	F	P	F	P	F	P	F	P
species	3	0.132	0.941	0.147	0.931	0.015	0.998	0.084	0.968	0.013	0.998	0.344	0.794
Temperature treatment	2	0.452	0.638	0.631	0.535	1.138	0.326	0.981	0.379	1.246	0.293	0.998	0.374
canopy	1	0.044	0.834	0.005	0.164	0.013	0.909	0.011	0.918	0.006	0.939	0.092	0.763
Temperature × species	6	1.521	0.181	1.576	0.164	1.595	0.160	1.436	0.211	1.067	0.390	1.304	0.266
Canopy × temperature treatment	2	1.124	0.330	1.468	0.236	0.545	0.582	0.673	0.513	0.133	0.876	0.509	0.603
Canopy × species	3	1.362	0.260	1.239	0.300	0.936	0.427	0.773	0.512	0.344	0.794	0.431	0.731

**Table 6:** General linear model of other fungal OTU richness based upon the rarefication of the observed species to 50 sequences

	DF	Richness metrics				Diversity metrics				Evenness metrics			
		Chao1		Observed species		Shannon		Simpson		Shannon		Simpson	
		F	P	F	P	F	P	F	P	F	P	F	P
Species	3	2.493	0.068	1.924	0.134	2.066	0.113	2.135	0.104	1.915	0.136	1.778	0.160
Temperature treatment	2	1.323	0.273	1.086	0.344	2.386	0.100	*3.559*	*0.034*	*4.411*	*0.016*	2.232	0.115
Canopy	1	*4.164*	*0.045*	*4.335*	*0.041*	*5.707*	*0.020*	*5.857*	*0.018*	2.624	0.110	0.881	0.351
Temperature × species	6	2.016	0.076	1.937	0.087	2.152	0.059	1.941	0.087	1.879	0.097	1.830	0.106
Canopy × temperature treatment	2	0.651	0.585	0.314	0.815	0.051	0.984	0.132	0.941	0.353	0.787	0.298	0.827
Canopy × species	3	*4.083*	*0.021*	*3.459*	*0.037*	2.063	0.135	1.178	0.314	1.462	0.239	0.416	0.661

Values matched in italics are statistically significant

## Discussion

In the present study, we investigated how projected increases in global temperatures will affect root ECM fungi of boreal and temperate tree species, and how this might interact with canopy structure. Host species [34, 35, 61], soil quality [31, 62], and canopy structure [17, 63] have all been previously reported to influence ECM fungal community composition. Studies that are able to control host species identity across broad geographic ranges have also showed climatic conditions to have a strong impact on ECM fungal community [33, 64, 65]. Similarly, in our study, all three experimental factors (host, temperature treatment, and canopy structure) were found to be important determinants of ECM fungal community structure.

Our initial hypothesis was that ECM fungal community response to increased temperature in a climate and ecosystem characteristic of the boreal-temperate ecotone is dependent on host species, with warming increasing the presence of characteristically temperate ECM fungal taxa on boreal host species. Our premise was that, in order to survive, boreal species growing at the warmer trailing edge of their range will switch their symbionts to taxa that are better adapted to higher temperature [25]. Fungi belonging to long-distance exploration type form rhizomorphs and are hydrophobic and efficiently transport nutrient and water [66] and longer distance exploration type fungal taxa increase across warming [27]. Although we found *Suillus* species on tree root, data from the current study did not support this hypothesis, as most of the most abundant taxa in our experiment (e.g., *Tomentella* or *Russulaceae*) or those that significantly increased at elevated temperature (e.g. *Laccaria*), equally colonized both temperate and boreal species. In the present study, the examined seedlings were growing under the experimental conditions for 3 years prior to sampling, and therefore, their fungal communities likely reflect experimental rather than nursery conditions. However, we cannot exclude survival of some nursery species on seedling root since data are not consistent showing both survival of some fungal species [67, 68] and shaping composition of ECM fungal communities after 2 years chiefly by environmental factors rather than pre-inoculation with different mycorrhizal fungi [69]. Taxa such as *Lactarius*, *Russula*, and *Tomentella* commonly occur in northern temperate ecosystems [reviewed by 38]. Those ECM species are host generalist, which is more common than ECM fungal affiliation towards specific tree species [70–73]. Thus, the absence of specific instances where temperate fungal species switched to roots of boreal species may be explained by the nature of *Russulaceae* or *Thelephoraceae* which are generalist ECM fungi and dominated in the experimental plots used in the current study. It has been observed that diversity of ECM fungal community is reduced in mixed forest stands [74]. Thus, the structure of diverse tree species used in our experiment could have also promoted lower ECM species diversity increasing proportion of the ECM fungal generalists. According to Carignan and Villard [75], cosmopolitan fungi are less sensitive to environmental changes than fungi restricted to specific site conditions. Also, Lankau et al. [76] found less deleterious effects of climate change on ECM relationships growing at the trailing edge of their geographic distribution, suggesting similar conclusions to Fisichelli et al. [11] that climate warming will not cause recession of boreal tree species, but rather, will result in a broadening of the transition zone in which boreal and temperate species exist together.

The most diverse taxa in our study, *Inocybe*, *Cortinarius*, and *Sebacina*, have been previously shown to be most diverse in colder climatic conditions, such as the Arctic [12, 37, 77]. Although 84 ECM OTUs were detected in the present study, most were not very abundant. Response to the environmental conditions administered in this study did not appear to be phylogenetically consistent, with OTUs belonging to the same genus responding differently to the same experimental treatments. Thus, apart from fungal generalists that have wide temperature optima, the diverse responses observed within taxa may indicate that these genera have different temperature optima. Switching from OTUs to another better adapted to changed condition within the same fungal taxa may be another reason why we did not observe temperate ECM fungi on boreal tree species. While this is somewhat in contrast with Tibbett et al. [78], Rygiewicz et al. [30], and López-Gutiérrez et al. [79], who found consistent genus-level responses to temperature, other global change experiments have also observed inconsistent phylogenetic responses, for example to elevated CO_2_ [80, 81].

The warming treatment appeared to affect the foraging type of ECM, which can be classified into “exploration types” based on their ability to form features such as extramatrical mycelia and rhizomorphs, which determine foraging range in the surrounding soil [66]. Examples of common exploration types include a contact exploration type which is characterized by a smooth mantle, few emanating hyphae and very short foraging distance, and a long-distance exploration type capable of forming rhizomorphs that can extend distances in the soil and intermediate type (e.g., medium smooth—forming smooth mantle and rhizomorphs). In our study, warming decreased medium-smooth and contact exploration types, such as *Russula* (OTU_44), *Tomentella* (OTU_6), and *Clavulina* (OTU_30). Deslippe et al. [27] and Jarvis et al. [31] also observed a negative influence of warming on medium-smooth, contact, and short exploration types. Fungi characterized as short and medium-smooth exploration types have a lower biomass of extramatrical hyphae and are thus able to regenerate more rapidly in response to an environmental disturbance [82]. Growth conditions and belowground C flux, however, enable the colonization of new absorptive roots with fungal communities that are affected by experimental conditions along with previously established ECM fungal species. Thus, stronger mycelial competitors become dominant over time [83]. While foraging types are often interpreted to reflect fungal nutrient uptake strategy [84], they may also reflect a trade-off in root colonization and competition strategies [83, 85]. In this light, the reduction we observed in the presence of ECM fungi that did not form extramatrical mycelia might indicate that they were weaker dispersers under the environmental conditions used in the present study. Given that short-contact exploration type fungi are more characteristic in high root density conditions [85], and more biomass is allocated to fine roots [6], increasing temperature in our experiment might also decrease the number of fine roots. Despite previously found results of increased participation of ECM fungi characterized with long-distance exploration type in temperate environments in contrast to boreal [64], domination of ECM fungi with short-distance exploration type in our experimental plots may be also the reason why our symbiont-switching hypothesis was not confirmed.

Previous global analyses found that both temperature and precipitation together were important factors affecting ECM fungal richness [36, 86]. In contrast, increases in ECM fungal OTU richness were not observed in temperate tree species in our study in response to temperature treatments, nor were decreases observed in boreal species. Diminishing richness of ectomycorrhizal species with different physiological and ecological functions may make the community more susceptible to perturbations [87–90]. In this regard, our results suggest that ECM fungi may be adaptable and tolerant to anthropogenic warming, at least up to a point. Additionally, our results may indicate that tree hosts switch their fungal symbionts [as was suggested by 25] to adjust to new conditions (e.g., warming and/or drying conditions) and that fungal community composition can be altered without alteration in richness. Such an adjustment is plausible when the local environment is rich in ECM fungal inocula. A shift in species richness is mainly driven by rare species that are more sensitive to unfavorable environmental conditions [36] or changes in soil attributes [63, 90]. The lack of differences in fungal richness observed in the present study, despite the richness in host species in experimental plots, may be due to dominance of the generalist species that were found on roots rather than specialist/rare species of ECM fungi. On the other hand, as suggested by Jarvis et al. [31] and Coince et al. [91], no difference in ECM fungal richness may be the result of insufficient saturation of the rarefied sampling curve and a deficiency in the identification of all the ECM fungal taxa inhabiting the root system of the tree species present in the experimental plots.

The higher energy influx in the open canopy was hypothesized to support a greater ECM richness. This hypothesis was not confirmed in our study, but a similar result was also reported in a tropical rainforest ECM community where it was interpreted as a decrease in the availability of local ECM inoculum supported by overstory trees [63]. However, the open canopy plots were established in near distance to the forest border on site where tree stumps were cut to the height less than 30 cm 1 year before the appropriate experiments stated [39]. Thus, the fungal inoculum may not be an issue. Given the linear relationship between gross primary productivity and carbon fluxes to both above and belowground plant organs [92], an increase in the supply of carbohydrates belowground should have promoted ECM richness in our experimental study. Reich et al. [18] demonstrated that, in contrast to the temperate species *Q. rubra*, warming negatively affect the physiology (net photosynthesis and growth) of the boreal species *P. glauca*. Additionally, they observed a neutral response to simulated warming in the boreal species, *B. papyrifera* and the temperate species, *P. strobus* [18]. When growing in an open versus a closed canopy, *P. glauca* and *Q. rubra* species responded differently to simulated warming [18]. ECM fungal richness in our studies did not correspond to changes in host photosynthesis. Interpreting this finding illustrates the complexity of predicting responses of mutualistic communities, since their responses are affected directly and indirectly by tree physiology as well as their own direct responses to environmental changes. Thus, the response of the ECM fungal community may be affected directly by specific experimental factors and indirectly by environmental conditions, both of which may alter the response of the ECM fungal community to host species.

## Conclusion

In the present study, we investigated the effect of projected increases in global temperatures on root-associated fungi of boreal and temperate tree species, as well as how canopy structure (open vs. closed) may interact with the potential impact of elevated soil temperatures. We hypothesized that ECM fungal richness would increase for temperate hosts and decrease for boreal hosts in warmed plots, and ECM fungal community composition in warmed treatments would be dominated by fungi found on temperate host plants. Additionally, we expected richness would increase in open canopy plots. In contrast to our initial hypothesis, no changes in ECM fungal richness were observed in response to simulated warming in either boreal or temperate tree species. While ECM richness has been shown to change over latitudinal gradients of climatic conditions [86], our data suggest that temperature changes in the range of 1–4 °C may exert a lesser impact on the diversity of root symbiotic fungi when plant species and precipitation are held constant. We did find, however, that canopy structure, experimental warming, and tree species affected the species composition of ECM fungi. In the future, additional detailed studies are warranted and necessary in order to provide additional information pertaining to the functional response of ectomycorrhizal fungi to different environmental drivers. It is plausible that long-term warming may change the soil mineralization rate, which in turn may lead to the selection of fungi with different abilities to uptake N and P. Whether these community changes buffer or further exacerbate changes in ecosystem function will depend on how the traits that determine response to nutrient availability are linked with other functional traits that affect ecosystem function [93]. Previous experiments have indicated that ECM community composition is affected by climatic factors while community function is dependent upon local soil conditions [94, 95]. Collectively, the data of the present study and previous studies suggest that further experiments will be required in order to more definitively understand the factors that determine the large-scale patterns in ECM community composition.

## Electronic supplementary material

ESM 1 (XLS 419 kb)
